# Assessing the readability and quality of online patient information for laser tattoo removal

**DOI:** 10.1007/s10103-024-04110-2

**Published:** 2024-07-17

**Authors:** Philippe Jean-Pierre, Ryan Scheinkman, Keyvan Nouri

**Affiliations:** University of Miami School of Medicine, Miami, FL USA

**Keywords:** Laser tattoo removal, Readability, Patient education, Patient safety, Skin of color

## Abstract

Just as tattoos continue to increase in popularity, many people with tattoos also seek removal, often due to career concerns. Prospective clients interested in laser tattoo removal may do research about the procedure online, as the internet increasingly becomes a resource to get preliminary health information. However, it is important that the online health information on the topic be of high quality and be accessible to all patients. We analyzed 77 websites from a Google search query using the terms “Laser tattoo removal patient Information” and “Laser tattoo removal patient Instructions” to assess this. The websites were evaluated for their readability using multiple validated indices and comprehensiveness. We found that websites had a broad readability range, from elementary to college, though most were above the recommended eighth-grade reading level. Less than half of the websites adequately discussed the increased risk of pigmentary complications in the skin of color clients or emphasized the importance of consulting with a board-certified dermatologist/plastic surgeon before the procedure. Over 90% of the websites noted that multiple laser treatments are likely needed for complete clearance of tattoos. The findings from our study underscore a significant gap in the accessibility and quality of online information for patients considering laser tattoo removal, particularly in addressing specific risks for patients with darker skin tones and emphasizing the need for consulting a board-certified physician before undergoing the procedure. It is important that online resources for laser tattoo removal be appropriately written to allow better decision-making, expectations, and future satisfaction for potential clients interested in the procedure.

## Introduction

Recently, tattoos have increased in popularity, with an estimated 32% of American adults having one [1]. Many regret their tattoos and seek removal, the most common reason being career concerns [2]. Laser removal is the most effective method for tattoo removal with the least side effects [3]. Similar to other dermatologic concerns, many prospective laser tattoo removal clients may seek preliminary information online [4]. The American Academy of Dermatology (AAD) recommends that all prospective laser tattoo removal clients consult a board-certified physician before the procedure for safety and best results [5]. Additionally, it is essential that online health information on the procedure mentions possible adverse events while maintaining a level of readability accessible to all patients [6, 7]. Thus, our study aimed to assess the quality and comprehensiveness of online patient information on laser tattoo removal along the aforementioned lines.

## Materials and methods

**Fig. 1:**
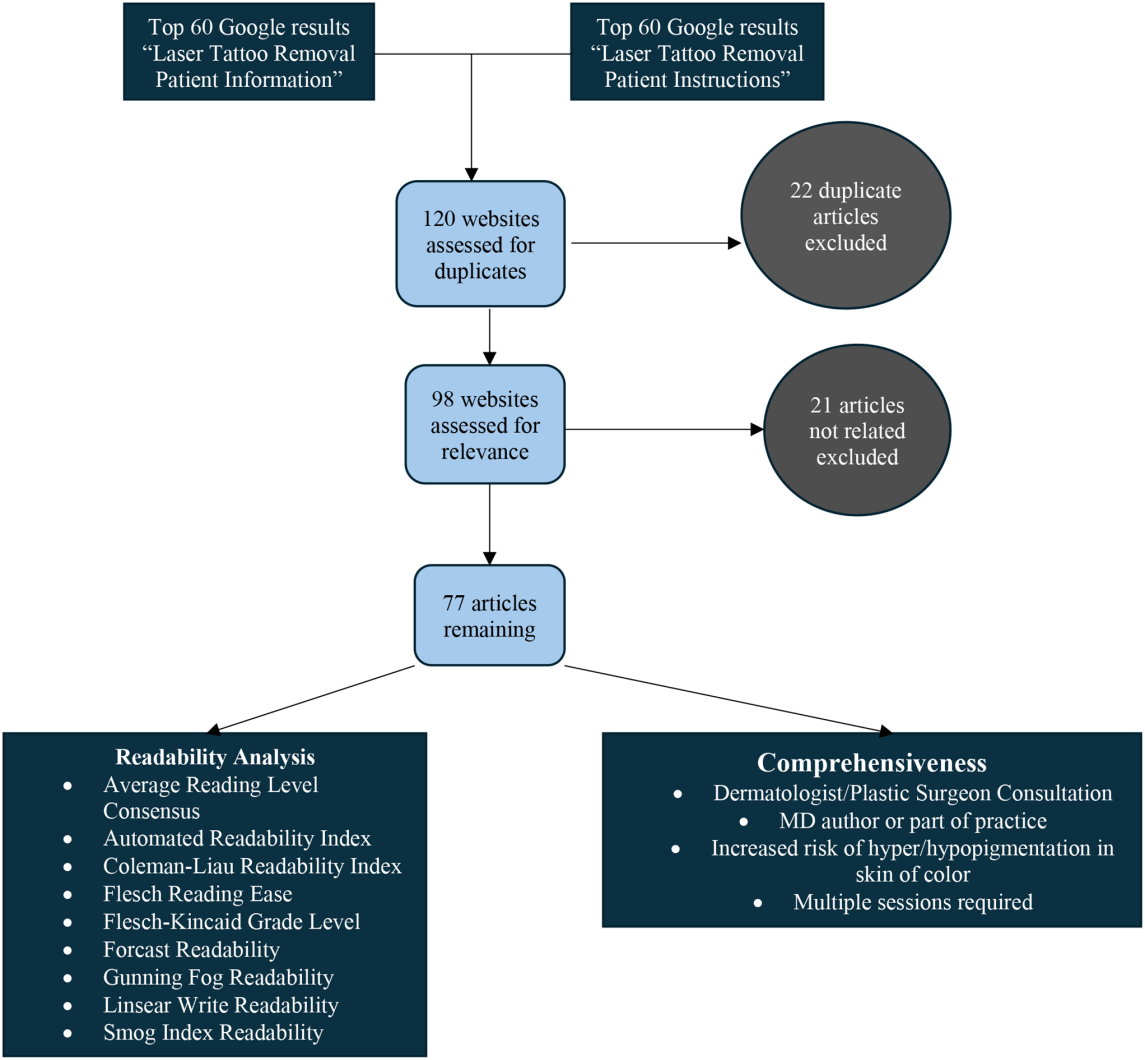
Curation of websites for laser tattoo removal information after exclusion criteria.

We performed a Google search using the search terms “Laser Tattoo Removal Patient Information” and “Laser Tattoo Removal Patient Instructions” using Google Chrome’s incognito mode. This mode hides previous browser data and user location, and we cleared cookies to decrease the potential for search biases. We screened the first 60 resulting links in each query for inclusion. Exclusion criteria included websites that did not have information on laser tattoo removal, were not in English, were sponsored links, and had duplicates. We then assessed the readability of the articles included using validated criteria. We also assessed whether the articles mentioned the necessity of multiple sessions, potential complications, special considerations for skin of color clients, and the importance of a consultation with a dermatologist or plastic surgeon. A flowchart of our methodology is shown in Fig. 1.

## Results

**Fig. 2:**
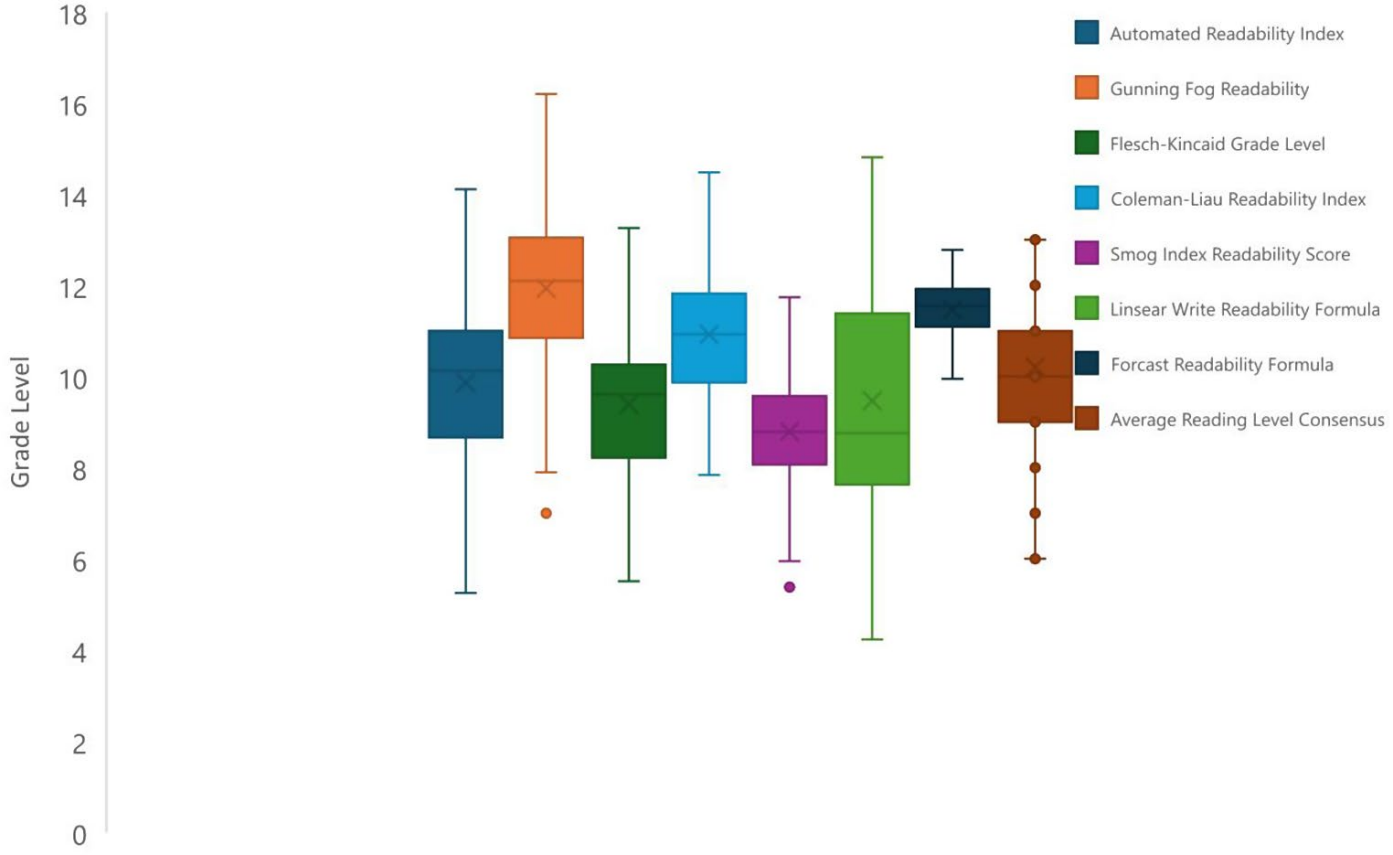
Readability Indices for 77 included websites

**Fig. 3:**
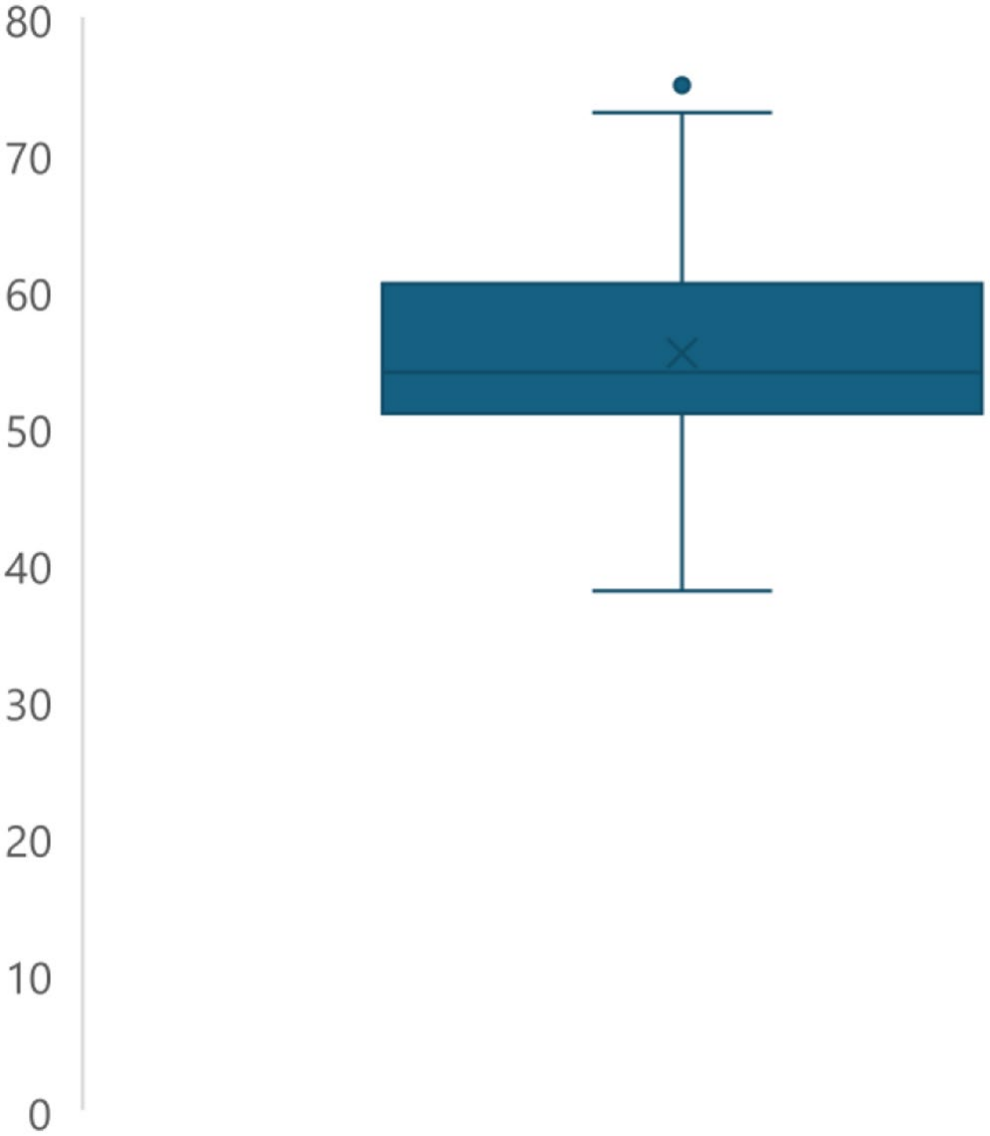
Flesch Reading Ease Scores for 77 included websites

The 77 webpages were assessed for readability in terms of approximate grade level using the Automated Readability Index, Gunning Fog Readability, Flesch-Kincaid Grade Level, Coleman-Liau Readability Index, Smog Index Readability Score, Linsear Write Readability Formula, and Forcast Readability Formula. These formulas were combined for the average reading level consensus calculated score that averages the seven previously mentioned scores and reports them as an integer grade level value. They were also assessed on a scale from 0 to 100 of increasing difficulty of readability using the Flesch Reading Ease scale. The readability results for the 77 webpages for Laser tattoo removal included in this study are reported in Fig. 2 (whiskers demonstrating minimums and maximums) for the 7 readability scales, and consensus scale used that correlated with approximate grade level and Fig. 3 for the Flesch Reading Ease scale (which is out of 100).

The results demonstrated that the patient information and instructions varied from just above elementary level (e.g., minimum of 4.23 for the Linsear Write Readability Formula) or middle school to college level (e.g., maximum of 16.2 for the Gunning Fog Readability scale) regardless of which readability scale was used. However, the mean readability scores were all within the high school grade range for the 7 scales used, which can be seen with standard deviations and the mean for the Flesch Reading Ease Scale in Table 1. The averages of all seven scales were above the 8th -grade reading level, combined with the general variability of the data, demonstrate that even though this data is intended for the general patient population, the reading level is more advanced than what is recommended for similar types of patient information (e.g., consent forms) by academic, professional, and governmental agencies [8, 9].

**Table 1:** Mean and standard deviation of readibility scores

Case	Mean	Standard Deviation
Automated Readability Index	9.86	1.82
Gunning Fog Readability	11.94	1.72
Flesch-Kincaid Grade Level	9.39	1.57
Coleman-Liau Readability Index	10.93	1.29
Smog Index Readability Score	8.79	1.23
Linsear Write Readability Formula	9.47	2.46
Forcast Readability Formula	11.47	0.62
Flesch Reading Ease	55.40	7.61
Average Reading Level Consensus	10.21	1.54

Additionally, less than half of the websites (43%) directly addressed complications associated with the procedure specifically for Skin of Color patients. Less than half of the sites (45%) recommended a consultation with a dermatologist or plastic surgeon before undergoing laser tattoo removal treatments. Only 53% of the websites had physicians with MD degrees as part of the patient care for the patients receiving laser tattoo removal. However, 90% of the websites did mention that multiple tattoo removal procedures would most likely be needed for optimal results (Table 2).

**Table 2:** Content On websites

MD - Part of Practice	Multiple Treatments	Skin of Color Complications	Dermatologist/Plastic Surgeon Consultation
53%	90%	43%	45%

## Discussion

The results of this study demonstrate the challenging readability of patient information and instructions for laser tattoo removal, reflecting the results of other studies analyzing dermatological diseases and procedures (e.g., Ezemma et al.’s analysis of patient materials for central centrifugal cicatricial alopecia) [10, 11]. Thus, this demonstrates a general trend in the readability of dermatological patient information above the recommended patient reading levels by professional and governmental bodies [8, 9]. Additionally, websites failed to adequately inform Skin of Color patients of the adverse effects of laser tattoo removal for hypopigmentation, hyperpigmentation, and scarring that disproportionately affect patients with darker skin tones [12]. Guidelines from the Centers for Disease Control and Prevention and the American Academy of Dermatologists recommend that patients consult a dermatologist before pursuing laser tattoo removal to minimize potential complication risk [5, 13]. However, of the 77 websites analyzed, fewer than half recommended consultation with a dermatologist or plastic surgeon, and only 53% of the webpages either were written by a physician with a Doctor of Medicine degree or had one as part of the patient care for the laser tattoo removal. Guidelines also recommend multiple treatments that are sufficiently temporally spaced to improve cosmesis and minimize complications, with the majority (90%) of the web pages analyzed recommending multiple-spaced treatments [5, 13]. Many of the webpages were for medical practices, with the highest medical provider being either a laser technician, nurse, or physician’s assistant as part of the patient care team, which poses a challenge to the potential validity of the information provided to the patients in addition to issues of readability.

One of the limitations of this study is the focus on English language websites. Studies on a combination of language and social barriers contribute to lower health literacy among Hispanic populations compared to other ethnic groups, with poorer health literacy associated with worse health outcomes (e.g., more hospitalizations) [14, 15]. Therefore, websites in Spanish that have appropriate readability are important for potentially improving health literacy and outcomes for Hispanic and Spanish-speaking patients. However, the authors of this study were not sufficiently fluent in Spanish to properly assess the characteristics assessed for this study, and future studies should focus on Spanish and other language websites.
